# Deep denoising for multi-dimensional synchrotron X-ray tomography without high-quality reference data

**DOI:** 10.1038/s41598-021-91084-8

**Published:** 2021-06-04

**Authors:** Allard A. Hendriksen, Minna Bührer, Laura Leone, Marco Merlini, Nicola Vigano, Daniël M. Pelt, Federica Marone, Marco di Michiel, K. Joost Batenburg

**Affiliations:** 1 Centrum Wiskunde and Informatica, Amsterdam, The Netherlands; 2 Swiss Light Source, Paul Scherrer Institute, Villigen, Switzerland; 3 Dipartimento di Scienze della Terra, Università degli Studi di Milano, Milan, Italy; 4 ESRF — The European Synchrotron, Grenoble, France; 5 Leiden Institute of Advanced Computer Science, Leiden Universiteit, Leiden, The Netherlands

**Keywords:** Imaging techniques, X-rays, Computational science, Software, Image processing

## Abstract

Synchrotron X-ray tomography enables the examination of the internal structure of materials at submicron spatial resolution and subsecond temporal resolution. Unavoidable experimental constraints can impose dose and time limits on the measurements, introducing noise in the reconstructed images. Convolutional neural networks (CNNs) have emerged as a powerful tool to remove noise from reconstructed images. However, their training typically requires collecting a dataset of paired noisy and high-quality measurements, which is a major obstacle to their use in practice. To circumvent this problem, methods for CNN-based denoising have recently been proposed that require no separate training data beyond the already available noisy reconstructions. Among these, the Noise2Inverse method is specifically designed for tomography and related inverse problems. To date, applications of Noise2Inverse have only taken into account 2D spatial information. In this paper, we expand the application of Noise2Inverse in space, time, and spectrum-like domains. This development enhances applications to static and dynamic micro-tomography as well as X-ray diffraction tomography. Results on real-world datasets establish that Noise2Inverse is capable of accurate denoising and enables a substantial reduction in acquisition time while maintaining image quality.

## Introduction

Synchrotron-based X-ray tomography is a powerful technique for investigating the internal structure of objects with applications in energy research, materials science, life sciences, and many other fields^1–3^. Thanks to the high photon flux available at synchrotrons, experiments can be performed at speeds and microscopic scales that push the envelope of scientific imaging^4,5^. In these experiments, a rotating object is located in the path of an X-ray beam, and its projection is measured on a detector. A representation of the object is reconstructed from a series of its projection images. The projection images typically suffer from noise, which carries over to the reconstructed images. Noise can be reduced by increasing the exposure time or photon flux, resulting in a higher dose. In many cases, however, dose and exposure time are limited by unavoidable experimental constraints, such as fast sample dynamics, radiation damage, and alteration of system properties during prolonged exposure to high-flux synchrotron X-ray beams^4,6–10^. In such experiments, accurate denoising of the reconstructed images is a central problem.

Among techniques for removing noise from reconstructed images, deep convolutional neural network (CNN)-based methods have shown strong results, often outperforming conventional state-of-the-art techniques^11–13^. A substantial subset of these techniques are known as post-processing techniques. Here, a reconstruction is first computed using a fast reconstruction algorithm and a CNN is used to post-process the reconstructed image. Other approaches, such as learned iterative techniques^13^ also exist, but are generally not as computationally efficient. Most proposed methods, however, require *supervised training* before they can be applied to the problem at hand. That is, the networks are trained by using paired examples of noisy input reconstructions and high-quality target reconstructions.

In practice, obtaining the set of paired noisy and high-quality reconstructions required for supervised training is a major obstacle^14^. First, obtaining low-noise reconstructions may not be possible, due to the aforementioned experimental constraints. Second, accurate pairing of two different datasets may require time-consuming manual labor to register the images, especially because the accuracy of registration directly impacts the output quality of the trained network^15^. Sub-pixel inaccuracies may cause blurring of the network output, for example. Finally, with fewer than 50 synchrotron facilities worldwide, beamtime is a scarce resource^16^, and acquiring additional measurements may simply be too expensive.

To sidestep the issue of obtaining training data, CNN-based denoising techniques have recently been proposed that do not require the acquisition of high-quality images^17–24^. However, many of these techniques rely on assumptions about the source of noise that are not correct in tomography, resulting in suboptimal denoising accuracy^25^. As a solution to these difficulties, we have proposed Noise2Inverse^25^, which is a post-processing technique specifically designed for tomography and related inverse problems. Using *self-supervised* training, the acquisition process is exploited to create pairs of noisy reconstructions from a single tomographic dataset. Although no high-quality reconstructions are presented to the network, training still produces a denoising CNN whose accuracy is comparable to supervised CNN training^25^. To obtain a denoised reconstruction, the trained CNN can be applied both to the noisy training reconstruction and to similar data that is acquired in the future. The Noise2Inverse method can be applied in the aforementioned cases where collecting a training set is found to be an obstacle.

To obtain a denoising CNN, the reconstructed training pairs must satisfy certain conditions, which we describe in this paper and refer to as the *Noise2Inverse conditions*. In previous work, analysis of the Noise2Inverse conditions was limited to single-slice (2D) parallel-beam tomography^25^, treating multi-slice (3D) tomographic datasets as a stack of independent 2D problems. In the synchrotron community, however, 3D tomography is routine and many important research questions can only be answered using more advanced imaging techniques that make use of additional information in the time or spectral domain of the signal^4–10,26–30^. In this paper, we extend the analysis of the Noise2Inverse conditions to such techniques, including micro-tomography (space), dynamic micro-tomography (time), as well as X-ray diffraction computed tomography (diffractogram, spectrum-like). Taking into account the Noise2Inverse conditions, we tailor training procedures to each of these techniques. Our description of the training procedures for these imaging techniques may serve as a template for other use cases. In addition, we discuss how the acquisition can be refined to optimize the results of Noise2Inverse denoising. Finally, we apply Noise2Inverse to real-world datasets, compare it to commonly used reconstruction techniques, and investigate the possibility of reducing acquisition time without loss of image quality.

This paper is structured as follows. First, we introduce the basics of CNN-based denoising. Next, we describe the Noise2Inverse method for single-slice (2D) parallel-beam tomography. Then, for each of the three domains (space, time, and diffractogram) and corresponding imaging technique, we describe a Noise2Inverse training procedure that exploits the acquisition process to obtain a denoising CNN. Next, we present results of the application of Noise2Inverse to real-world datasets of each of these techniques, and conclude with a discussion.

## CNN-based denoising

**Figure 1:**
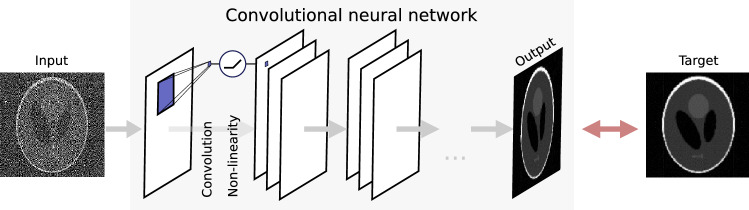
A prototypical convolutional neural network (CNN) in a supervised training arrangement for denoising. The CNN is composed of multiple layers. To compute an image in the next layer, images in the previous layer are convolved with a small filter (depicted in blue), after which a non-linear function is applied. To train a CNN to perform denoising, the output image is compared to a target image, and the convolution filters are updated accordingly.

This section briefly introduces convolutional neural networks (CNNs) for denoising. For a more detailed overview, we refer the reader to^31,32^. CNNs are usually organized in layers: the input image is copied into the first layer, the output image is the final layer, and computations are performed in the intermediate layers. The CNN computes convolutions that are parameterized by thousands to millions of small filters (typically $3 \times 3$). In each intermediate layer, the images in the previous layer are convolved with these filters, after which a pixel-wise non-linear function is applied. A common non-linearity is the ReLU function, which is the identity for positive arguments and zero otherwise^33,34^. A prototypical CNN is illustrated in Fig. 1. The input to the network can consist of multiple 2D channels, for example when the input is a color image. Similarly, the intermediate layers and output may consist of multiple channels—regardless of the number of channels in the input.

The network can be prepared to perform denoising by supervised training. During training, the network is presented with noisy input images and noise-free target images from a training dataset. On each image pair, the parameters of the convolutions are optimized to minimize the training loss, i.e., the difference between the output and target image. The training loss is commonly quantified by the mean square error^35^ and minimized using stochastic gradient descent^36^.

If the number of parameters of a CNN is large compared to the amount of training data, there is a risk of overfitting. In such situations, the network starts to fit features particular to the training data which causes its accuracy to suffer on its intended use case^14^. A practical remedy is to monitor the results of the network on a separate validation dataset, and perform early stopping when the results start deteriorating. Early stopping may not be necessary, however, if the number of training pixels vastly exceeds the number of CNN parameters^35^.

In tomography, the reconstructed data is often volumetric rather than a single 2D image. In the volumetric case, it has been observed that image quality can be improved by using 3D CNNs instead of 2D CNNs^37^. Here, the input and output are a 3D volume, and intermediate computations are performed using 3D convolutions. 3D CNNs, however, impose computational demands that can be prohibitive in practice. As a solution to this problem, 2.5D CNNs have been proposed. These networks can often achieve image quality similar to 3D CNNs at a reduced computational cost^37^. We refer to a 2D sub-image of a 3D volumetric dataset as a *slice*. The input to a 2.5D CNN is a stack of 2D slices, and its output is a 2D slice. In addition to the current input slice, the network input consists of context slices located above and below the current slice. This technique is applied to 3D tomographic datasets in section “Noise2Inverse for synchrotron X-ray tomography”.

As described before, the collection of paired image data can be a major obstacle to the use of supervised training. In the next section, we describe how a denoiser can be trained without such a training dataset.

## Methods

In this section, we discuss the Noise2Inverse method. First, we summarize the procedure for single-slice (2D) parallel-beam tomography and describe the conditions under which Noise2Inverse training is possible^25^. Next, we describe how Noise2Inverse can be extend in space, time, and spectrum-like domains so that it can be applied to imaging techniques in common use at synchrotron facilities.

### Noise2Inverse for single-slice parallel-beam tomography

**Figure 2:**
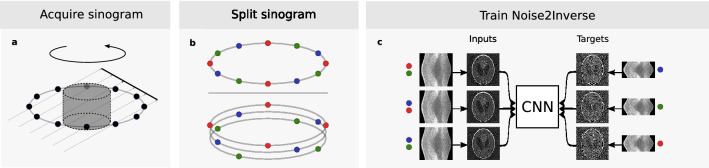
Noise2Inverse for single-slice parallel-beam tomography. (**a**), A sinogram is acquired of a rotating object. (**b**), The sinogram is split into sections (red, green, and blue) in the domain of the rotation angle. (**c**), The input and target images for training the network are reconstructed from distinct sections of the sinograms (indicated by the red, green, and blue dots). The input and target sections cover $\frac{2}{3}$ and $\frac{1}{3}$ of the sinogram, respectively.

In single-slice parallel-beam tomography, a rotating object is located in the path of a planar beam, and its projection is measured on a line detector, as displayed in Fig. 2. A 2D image, called the sinogram, is collected from readouts at a series of angles. From the sinogram, a 2D image (slice) of the object can be computed using a reconstruction algorithm^38^.

The Noise2Inverse procedure for parallel-beam tomography was comprehensively introduced in^25^. First, the acquired sinogram is split into multiple *target sections* in the domain of the rotation angle. Each target section has a matching *input section* that covers the remainder of the sinogram. As displayed in Fig. 2b, the target sections split the sinogram in such a way that adjacent measurements in the angular domain are in different target sections. During CNN training, the input and target images are reconstructed from the corresponding sections of the sinogram. The CNN thus learns to map noisy reconstructed images to each other. The training process is displayed in Fig. 2.

After training, the reconstructed images are post-processed using the CNN. In this paper, we propose a modification to the post-processing step described in^25^. There, the CNN was applied to reconstructions of the input sections separately, resulting in several denoised intermediate images, of which the average formed the output of the algorithm. In this work, however, the output is computed by directly applying the CNN to the reconstruction of the full sinogram. Empirically, this modification consistently improves results^19^, although no theoretical explanation for its success has yet emerged.

To train a denoising CNN, the Noise2Inverse method requires that the reconstructed training pairs satisfy two conditions, which we refer to as the **Noise2Inverse conditions**:

1. The noise in the reconstructed input image is statistically independent from noise in the reconstructed target image.
2. Every subset of the measurement is used in the reconstructed target images equally often.

The Noise2Inverse conditions are satisfied in the case of single-slice parallel-beam tomography. The noise in the reconstructed input and target images is independent (“uncorrelated”), because the images, and thus the noise, are reconstructed from distinct input and target sections of the sinogram. Because target sections from all parts of the sinogram are used during training, the second requirement is satisfied as well. This prevents biasing the result towards a specific subset of the measurements.

Formally, the first condition makes it possible to write the training loss as the sum of two terms. Consider a noisy measurement and its corresponding unknown noise-free sinogram. Split the measurement into *J* target sections and denote by $x_{j, \text {noise-free}}$ the reconstruction of the *j*th target section of the noise-free sinogram. Denote by $x_{j, \text {noisy}}$ the reconstruction of the *j*th target section of the noisy measurement and by $x_{\cdot \ne j, \text {noisy}}$ the corresponding input reconstruction. The training loss can then be decomposed as a sum of two terms^25^

1 $$\begin{aligned} \underbrace{ \sum _{j=1}^{J} \Vert \text {CNN}(x_{\cdot \ne j, \text {noisy}}) - x_{j, \text {noisy}} \Vert ^{2}_{2} }_{{\text {Noise2Inverse training loss}}} {\mathop {=}\limits ^{\mathbb {E}}} \sum _{j=1}^{J} \underbrace{ \Vert \text {CNN}(x_{\cdot \ne j, \text {noisy}}) - x_{j, \text {noise-free}} \Vert ^{2}_{2} }_{\text {Loss w.r.t. noise-free reconstructions}} + \underbrace{ \Vert x_{j, \text {noise-free}} - x_{j, \text {noisy}} \Vert ^{2}_{2} }_{\text {Variance of the noise}}, \end{aligned}$$

where ${\mathop {=}\limits ^{\mathbb {E}}}$ indicates that the equality holds on average over the statistical distribution of the noise and the imaged objects. On the right hand side, the first term represents the difference between the CNN output and the noise-free reconstructions, and the second term represents the variance of the noise in the reconstructed images, which does not depend on the CNN. Minimizing the Noise2Inverse training loss therefore minimizes the loss with respect to the noise-free reconstructions, which is the goal of denoising.

Equation (1) holds in combination with any linear reconstruction algorithm. Most common reconstruction algorithms in synchrotron tomography fall into this category, e.g., FBP, GridRec, and SIRT^38–40^. An additional requirement is that the measurement noise is zero-mean. This is typically satisfied by sources of noise in X-ray synchrotron tomography, such as Poisson or additive Gaussian noise. An example of noise that is not zero-mean is salt and pepper noise, which randomly corrupts pixels by setting them to zero or a fixed high value.

Intuitively, Noise2Inverse and other self-supervised methods rely on the ability of CNNs to find correlations between images. During training, the content of both the input and the target reconstruction is determined by the structure of the object—resulting in a strong structural correlation between the images—and the noise, which is uncorrelated. Therefore, the network is rewarded when it makes a prediction using the structure of the object. If it picks up a correlation based on the noise, it may be punished when this spurious correlation is absent in later iterations. During training, the network thus learns the statistical distribution of noiseless reconstructions. This distribution encompasses all features of the reconstructed images that are not related to the measurement noise. Therefore, when systematic imperfections in the measurement carry over into the reconstructed images, the network will not learn to remove them. This can occur, for instance, when the angular sampling of the full sinogram has insufficient resolution, as described in^25^.

If the Noise2Inverse conditions are not satisfied, then the output of the trained CNN can be sub-optimal. First, if the noise in the reconstructed input and target images is not independent, then the CNN will learn to reintroduce noise in its output. This can happen when the input and target sections of the measured data partially overlap, for instance. Second, when not every subset of the measurement is used in the target image equally often, then the CNN may be trained to overemphasize reconstruction of certain sections of the measured data, resulting in biased output.

### Noise2Inverse for synchrotron X-ray tomography

In this section, we propose Noise2Inverse training strategies tailored to imaging techniques in common use at synchrotron facilities. We discuss extensions in the spatial domain (3D micro-tomography), time domain (dynamic micro-tomography), and spectrum-like domain (X-ray diffraction tomography). In addition, we discuss how the training strategies satisfy the Noise2Inverse conditions.

#### Static 3D micro-tomography

**Figure 3:**
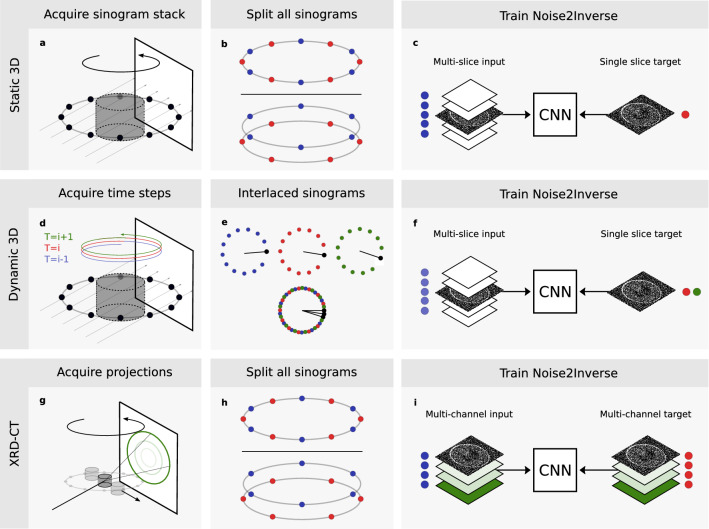
Noise2Inverse training procedures for several imaging techniques. (**a**-**c**), Static 3D micro-tomography. Acquisition produces a stack of sinograms (**a**), each of which is split in the angular domain (**b**). A 2.5D-CNN is supplied with the current input slice and several context slices (shown in white) reconstructed from one part of the sinogram (blue dot). The target slice is reconstructed from a different part of the sinogram (red dot). (**d**-**f**), Dynamic micro-tomography of an evolving process. Scans of multiple time steps are acquired (**d**). With interlaced acquisition, the angular sampling of each time step is slightly displaced with respect to the previous time step (indicated by the “clock’s hands”) (**e**). Denser angular sampling is achieved by combining the sinograms of multiple time steps (**e**). A 2.5D-CNN is trained on time steps where the dynamic process has not yet started (**f**). The input is a reconstruction of a single time step (blue dot), and the target is reconstructed from the sinograms of the other time steps (red and green dots). (**g**–**i**), X-ray diffraction computed tomography (XRD-CT). A pencil beam probes a rotating object that is moved sideways in steps (**g**). Diffraction of the beam gives rise to rings on the detector (in green). Azimuthal integration along rings at different radii yields several sinograms for each slice of the object. These sinograms are split in the domain of the rotation angle (blue and red dots) (**h**). From these sinograms, a multi-channel reconstruction is computed, representing the diffractogram of the object (in shades of green) (**i**). Training is performed with multi-channel inputs and multi-channel targets, with inputs and targets reconstructed from distinct parts of the sinograms (blue and red dots).

In 3D micro-tomography, the beam is measured on a 2D detector instead of a line detector. In the parallel-beam case, this problem can be viewed as a stack of single-slice problems. Therefore, the strategy from the previous section could be pursued, using reconstructed image pairs from the entire stack to train a single 2D CNN^25^. As discussed in section “CNN-based denoising”, however, the image quality can be further improved by using a 2.5D CNN^37^. This network’s input consists of context slices located above and below the current slice, as illustrated in Fig. 3c.

The Noise2Inverse strategy can be used with 2.5D CNNs. After acquisition of a stack of sinograms, we propose that each sinogram is split in the angular domain to obtain target sections and complementary input sections. In each training iteration, the target image is a single slice reconstructed from a target section. The input consists of an input slice and context slices reconstructed from the corresponding input section. The procedure is illustrated in Fig. 3. This strategy preserves the Noise2Inverse conditions, as the noise in the input and target is reconstructed from distinct sections of the sinograms, and each part of the sinogram is used as target section.

#### Dynamic micro-tomography

In dynamic tomography, a full scan of a dynamically evolving process is made at several time steps. In a single time step, movement of the object can result in motion artifacts, severely degrading the quality of the reconstructed image. To prevent such artifacts, the ideal scan time of a time step is bounded by the speed of the evolving process^4,6^. Additionally, the number of projection images per time step may be limited by the maximum frame rate of the detector. Therefore, both the exposure time and the number of projection images per rotation may be limited, and a reconstruction of a single time step may suffer from sparse angle artifacts^41^.

When performing dynamic tomography with angular undersampling, Noise2Inverse benefits from interlaced angular sampling^42^. Here, the angular sampling at each time step is displaced from the sampling at the previous time step. By combining sinograms of multiple time steps, this technique enables denser angular sampling to be achieved, as illustrated in Fig. 3e. From the combined sinogram, an accurate reconstruction can be computed, possibly even avoiding motion artifacts where and when the object is static. As discussed in Supplementary Note S1, it is likely that a fast dynamic scan uses interlaced angular sampling, either deliberately or as a consequence of minimal mechanical inaccuracies.

Typically, the dynamic process does not start immediately during the acquisition. This creates a window of opportunity to train a CNN on the first few time steps. Here, interlaced sampling mitigates the effects of undersampling, and motion artifacts are avoided, since the dynamic process has not yet started. We propose that the sinogram stacks of these time steps are combined into a single sinogram stack. The sinogram stack is subdivided such that each input section covers a single time step, and the corresponding target section covers the remaining time steps. During training, the input and target are reconstructed from the input and target sections. This process is displayed in Fig. 3d-f, where three time steps are combined into a single sinogram stack, and two time steps are used to reconstruct the target image in a round-robin fashion. The number of time steps should preferably be chosen such that the interlaced sampling distributes the angles of the projection images evenly over a 360° arc. As in the static 3D case, 2.5D CNN training can be used. After training, the network is applied to each reconstructed time step to obtain a sequence of denoised reconstructions. A similar approach has been described for dynamic magnetic resonance imaging (MRI)^21^.

This training strategy preserves the Noise2Inverse conditions. The noise in the reconstructed input and target images is independent as the images have been reconstructed from distinct time steps. The second property is satisfied, as each time step occurs in the target images equally often. Note that in contrast to the previous cases, the target is reconstructed from *more* measurements than the input, since the target is computed using multiple time steps. This enables the target reconstructions to achieve denser angular sampling and causes the network to mitigate sparse angle artifacts.

#### X-ray diffraction computed tomography

X-ray diffraction tomography (XRD-CT) makes it possible to non-destructively identify the crystallographic phases that compose a material, using the principles of X-ray powder diffraction^26,27^. As illustrated in Fig. 3g, the acquisition proceeds by probing a rotating object with a pencil beam, repeating the acquisition as the object is moved sideways in steps. The diffraction signal produced by the sample is recorded on a 2D detector, on which rings correspond to the scattering angle of the beam. By azimuthal integration along these rings, sinograms are computed for each scattering angle, forming 2D images indexed by the rotation angle and the sideways translation of the object. Performing the azimuthal integration with different radii yields several sinograms for one 2D horizontal slice of the object. From these sinograms, a multi-channel reconstruction is computed where each channel corresponds to a radius of the azimuthal integration. For each voxel in the 2D slice, these channels form a diffractogram, which is a non-spatial dimension analogous to a spectrum.

The Noise2Inverse strategy for X-ray diffraction tomography exploits the interdependence of the object in the domain of the diffractogram (across channels). The diffractogram at a location is determined by the specific crystallographic phase of a material at that location. Hence, if a material in a specific phase is present at multiple locations, its diffractogram—the values in the multi-channel reconstructed image—must correspond. The accuracy of denoising can be improved by exploiting this interdependence.

During Noise2Inverse training, we propose to split the obtained sinograms in the domain of the rotation angle to obtain input and target sections. Multi-channel reconstructions are computed from the input and target sections of the sinograms, which are used as input and target during training. This training strategy satisfies the Noise2Inverse conditions, as the input and target images are reconstructed from distinct sections of the sinograms, and the target sections cover the full sinogram.

In summary, Noise2Inverse can be adapted to make use of additional information in space, time, and spectrum-like domains. This enables its application to several imaging techniques in use at synchrotron X-ray facilities. In the case of static 3D micro-tomography, the 2D network input is extended with context slices to utilize 2.5D CNN training. In the case of dynamic 3D micro-tomography, the effects of sparse angular sampling are mitigated by reconstructing the target image from a combined sinogram of multiple time steps.

In the case of X-ray diffraction tomography, multiple channels represent the diffractogram of each voxel in a 2D slice of the object—not its vertical direction—, and thus serve a different purpose than in the previous cases.

## Results

We applied the Noise2Inverse method to datasets acquired at two synchrotron beamlines. In particular, we compared the method to common reconstruction algorithms on a static and a dynamic micro-tomography dataset from the TOMCAT beamline at the Swiss Light Source (SLS). In addition, we investigated the possibility of accelerating the acquisition process using an X-ray diffraction tomography (XRD-CT) dataset from the ID15A beamline at the European Synchrotron (ESRF). The acquisition procedure for each dataset is described in Supplementary Note S1.

On each dataset, CNN training was performed using the same parameter-efficient mixed-scale dense network architecture (MS-D)^43^ and the same hyper-parameters. Differences between the Noise2Inverse training procedures on each dataset are summarized in Table 1 and described in more detail in Supplementary Note S2.

**Figure 4:**
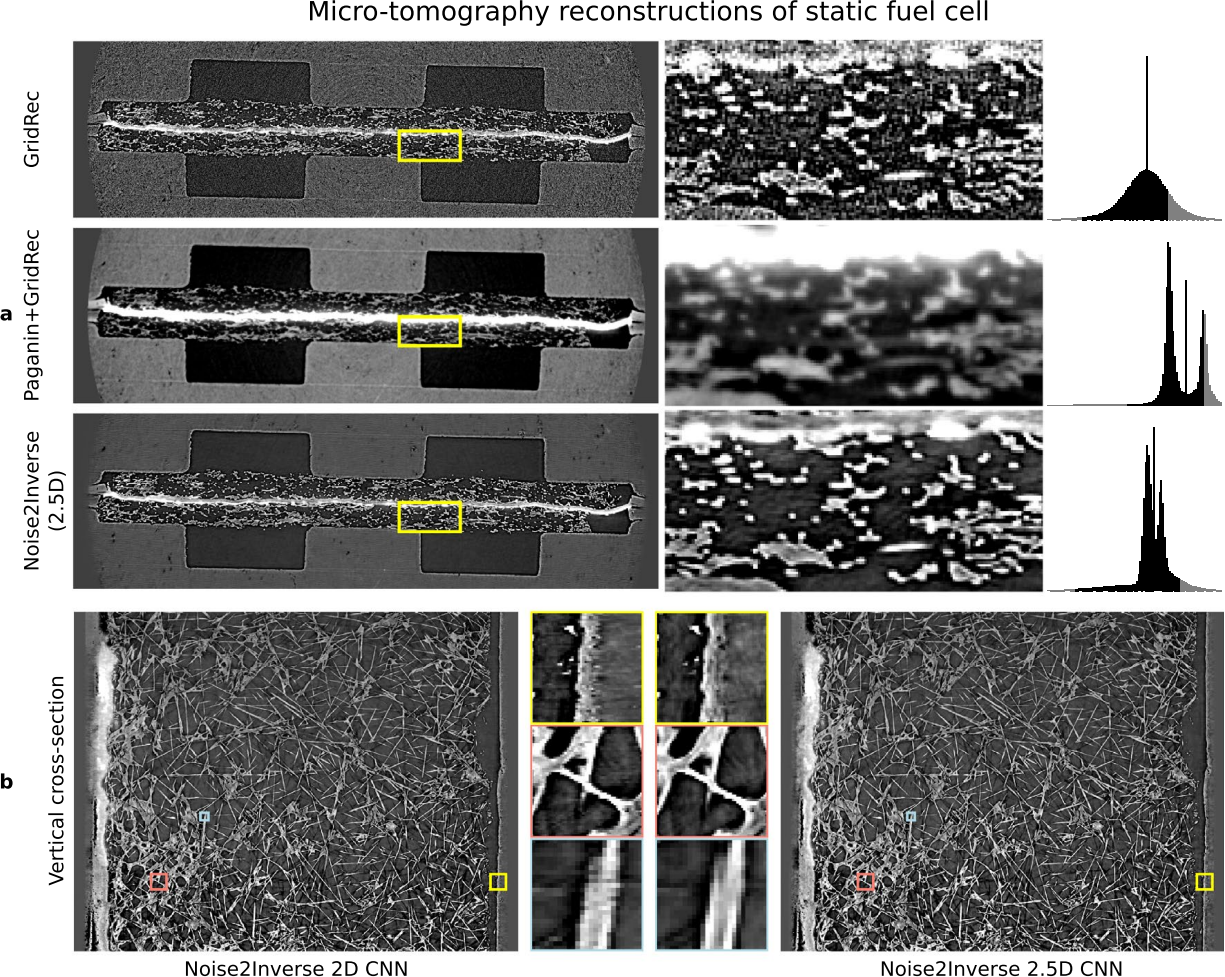
Comparison of reconstructions of a fuel cell using GridRec, Paganin+GridRec, and Noise2Inverse. (**a**), Reconstructions of the static fuel cell. The yellow region of interest is magnified in the windows on the right. Gray level ranges have been adjusted to improve contrast and are indicated by the shaded region in the histograms on the right. (**b**), A comparison of Noise2Inverse with a 2D CNN and a 2.5D CNN on a vertical cross-section of the reconstruction. The magnified regions of interest in the middle reveal discontinuities in the 2D CNN reconstruction.

**Static 3D micro-tomography** A static 3D micro-tomography dataset of a fuel cell was acquired. This dataset was part of a set of acquisitions aimed at imaging the water dynamics in the fuel cell. During operation, fuel cells generate water as a by-product. Suboptimal water management is currently a major limiting factor to sustained performance of the fuel cell at high current densities^6^. Accurate imaging of the water dynamics can inform improvements to the fuel cell design. This static dataset contained no water; a dynamic dataset of the operating fuel cell is discussed below.

We compared the Noise2Inverse reconstruction to GridRec reconstructions^39^ with and without additional preprocessing. The preprocessing was performed using Paganin phase retrieval^44^, and we refer to resulting reconstructions as Paganin+GridRec. For single material objects and monochromatic radiation, the Paganin algorithm^44^ can be used to preprocess the projection images prior to tomographic reconstruction to obtain quantitative results free of edge-enhancement artifacts. In the synchrotron community, thanks to its robustness, the Paganin algorithm is also used when not all assumptions are strictly satisfied as a tool to boost contrast and decrease noise in tomographic reconstructions. In both static and dynamic experiments with polychromatic radiation and a multi-material object, the parameters for the Paganin algorithm were chosen so as to maximize contrast while limiting the degradation of spatial resolution. The sinogram stacks were computed using standard dark-field, flat-field, and log-correction from the raw projection data for the GridRec and Noise2Inverse reconstructions. In the Paganin+GridRec reconstruction, the dark-field and flat-field corrections were applied before Paganin phase retrieval, and the log-correction was applied afterwards. We emphasize that the GridRec and Paganin+GridRec reconstructions were computed from exactly the same measured data as the Noise2Inverse reconstruction. No additional measurements were acquired for the Noise2Inverse reconstructions.

The results are shown in Fig. 4. The measurement noise was carried over into the GridRec reconstruction, and the Paganin+GridRec reconstruction was blurred. Image features in the Noise2Inverse reconstruction could be more easily distinguished, and the reconstruction did not suffer from noise or blurring. In addition, we trained a 2D CNN to illustrate the effect of not taking into account additional 3D information using a 2.5D CNN. Panel (**b**) displays a vertical cross-section of the resulting reconstruction that allows comparing between Noise2Inverse with a 2D CNN and with a 2.5D CNN. On this vertical reconstruction, we see that the 2D CNN reconstruction suffers from slight vertical discontinuities that are not present in the 2.5D CNN. The 2D CNN was not much faster to train than the 2.5D CNN.

**Figure 5:**
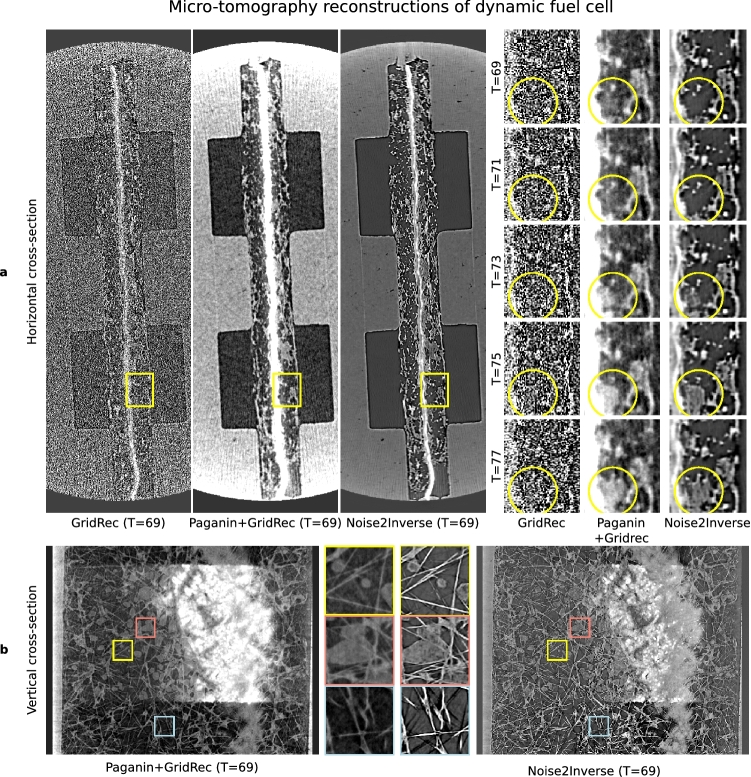
Comparison of reconstructions of an operating fuel cell using GridRec, Paganin+GridRec, and Noise2Inverse. (**a**), Reconstructions of the fuel cell in operation. The yellow region of interest is magnified in the windows on the right, and shows the formation of a water bubble over several time steps. (**b**), A vertical cross-section of the dynamic reconstruction is displayed with magnified regions of interest shown in the middle.

**Dynamic 3D micro-tomography** A dataset was acquired of a fuel cell that was in operation. The speed of the water dynamics made it necessary to acquire a single time step 10 times faster than in the static case, resulting in a highly restricted exposure time and a restricted angular sampling frequency—due to the maximum frame rate of the detector. During the acquisition, the object was continuously rotating at a constant speed. As described in Supplementary Note S1, we observed a small deviation in the rotation speed as a result of a minor mechanical inaccuracy. This caused each time steps’ 300 projection angles to be slightly displaced with respect to the previous time step, resulting in interlaced sampling. Training was performed on the first $3.6 \hbox { s}$ of the $18\hbox { s}$ acquisition (36 out of 180 time steps).

A comparison of GridRec, Paganin+GridRec, and Noise2Inverse can be found in Fig. 5. It shows a reconstruction of a horizontal slice, and several time steps of a magnified region of interest, in which the formation of a water bubble takes place. The GridRec reconstruction suffered severely from noise carried over into the reconstruction, and the Paganin+GridRec reconstruction was blurred, although the water dynamics were discoverable. The Noise2Inverse reconstruction was substantially sharper, and the water dynamics in the magnified views were clear. In panel (**b**), several crops show the difference in blurring between Noise2Inverse and Paganin+GridRec on a vertical cross-section.

**XRD-CT** An X-ray diffraction tomography dataset was acquired of an archaeological ceramic, whose fragments are kept at the University of Milan. The acquisition resulted in a dataset containing 3 horizontal slices with 11 channels each. Acquisition of each slice took 20 min. Two noisier datasets were obtained by applying synthetic noise to the sinograms. These were estimated to correspond to a virtual acquisition time of $70\%$ and $20\%$, respectively, as described in Supplementary Note S3. The relative variance of the noise of the synthetic datasets is displayed in Fig. S1.

**Figure 6:**
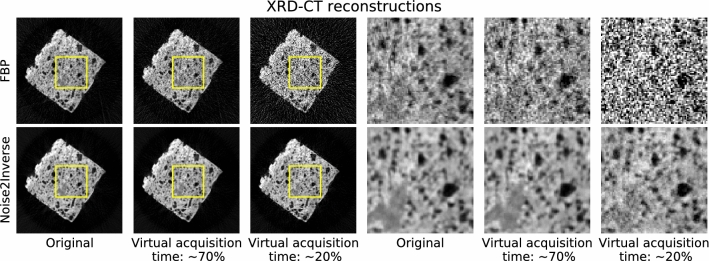
X-ray diffraction tomography reconstructions of a single channel of a single slice of a ceramic fragment. The leftmost column shows the reconstruction of the originally acquired data, and the next two columns show reconstructions with synthetic noise. The rightmost three columns show magnifications of the yellow region of interest.

The results are shown in Fig. 6. As the virtual acquisition time was decreased, the quality of the FBP reconstruction suffered due to measurement noise that was carried over into the reconstruction. Compared to the FBP reconstructions, the degradation in image quality of the Noise2Inverse reconstructions was substantially less severe.

### Training time, intermediate results, and overfitting

Differences of Noise2Inverse training between the three previous experiments are summarized in Table 1. The training times were realized on a system with 192 GB of RAM and four Nvidia GeForce GTX 1080 Ti GPUs. The reported training times are indicative, and are specific to the hardware. For instance, a networked storage bottleneck caused training on the dynamic dataset to take twice as long as on the static dataset, even though a similar number of training iterations was spent on both datasets.

Although training times were substantial for all three datasets, we note that training time did not increase linearly with the size of the training dataset. For instance, the dynamic dataset was 10, 000 times larger than the XRD-CT dataset, yet training time took less than 30 times longer. An important factor is that training time dominates reconstruction time: the static fuel cell took $20 \hbox { h}$ to train, but inference took only $5 \hbox { min}$ for instance. As shown in Table 1, the CNNs had roughly the same number of parameters for all three datasets. That may be the reason that the number of required training iterations does not scale with the size of the dataset, but reaches a point of diminishing returns. Therefore, training does not necessarily take much longer for larger datasets.

The number of training iterations was fixed in advance for each experiment. On intermediate results on the XRD-CT dataset, we observed that most of the improvement occurred in the first $20\%$ of training, in which all noise was removed, but the images were slightly blurred. Small details evolved in the remaining $80\%$ of training time. In^25^, it is reported that prolonged training can obtain some improvement in image metrics. In our experience, however, training for longer periods had no substantial influence on image quality. Because no ground truth data was available, changes in the output of the network were difficult to quantify. On the XRD-CT dataset, an additional training session was continued for ten times as many iterations (300,000). As training progressed, no reintroduction of noise into the reconstructed images was observed, and some small details were better resolved, indicating that overfitting was unlikely to occur.

**Table 1:** Details of Noise2Inverse training on the static and dynamic micro-tomography datasets of a fuel cell and X-ray diffraction tomography dataset of a ceramic. The relative section size reports the ratio of the size of the input section relative to the target section. The size of the training volumes varies widely, whereas the number of parameters in the CNN is stable. Reported training times are indicative, and are specific to the hardware.

	Static fuel cell	Dynamic fuel cell	XRD-CT ceramic
**Noise2Inverse**			
Channels (input, target)	11, 1	11, 1	11, 11
Relative section size (input : target)	1 : 1	1 : 5	2 : 1
**Size**			
Sinograms (32-bit float)	$6.3 \hbox { GB}$	$342 \hbox { GB}$	$9.2 \hbox { MB}$
Training volume size (voxels)	$6.86 \cdot 10^{8}$	$2.47 \cdot 10^{10}$	$2.46 \cdot 10^{6}$
CNN Parameters	$5.48 \cdot 10^{4}$	$5.48 \cdot 10^{4}$	$5.60 \cdot 10^{4}$
**Duration**			
Training iterations	220,000	171,600	30,000
Training $+$ Reconstruction duration	$\sim 20$ hours	$\sim 43$ hours	$\sim 90$ minutes

### Comparison to total-variation minimization

In this section, Noise2Inverse is compared to a traditional iterative reconstruction approach. We implemented Total-Variation Minimization (TV-MIN) using the Chambolle-Pock algorithm^45^. For each dataset, we used 500 iterations and determined the optimal regularization parameter $\lambda$ visually, as described in Supplementary Note S4. Reconstructions for several values of the regularization parameter are displayed in Fig. S2. The TV-MIN reconstruction was performed on a single slice of the reconstruction and did not take into account additional space, time, or diffractogram information that was present in the static micro-tomography, dynamic micro-tomography, or the XRD-CT dataset, respectively.

**Figure 7:**
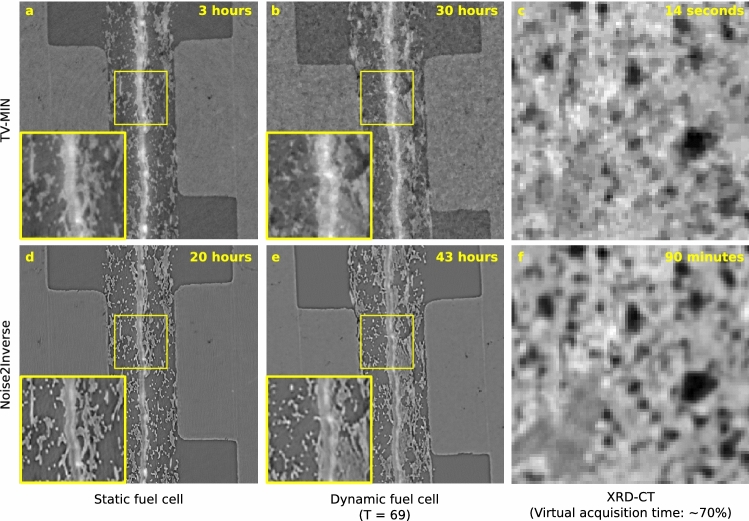
Comparison between Total-Variation Minimization reconstructions (**a**-**c**) and Noise2Inverse (**d**-**g**) reconstructions of the static and dynamic micro-tomography datasets of a fuel cell and X-ray diffraction tomography dataset of a ceramic. The displayed regions correspond to the reconstructions displayed in Figs. 4, 5, and 6. Magnifications of the yellow regions of interest are displayed in the lower-left corner of the panels. In the top right corner of each panel, an indication of the reconstruction time of the full dataset is given using four Nvidia GeForce GTX 1080 Ti GPUs. The indicated time was realized for the Noise2Inverse reconstruction and estimated for the TV-MIN reconstruction.

An indication of the reconstruction time is given in the top right corner of each panel in Fig. 7. For the Noise2Inverse reconstruction, the realized reconstruction time is reported. For the TV-MIN reconstruction, an idealized reconstruction time is calculated by performing a single-slice reconstruction on a single GPU. To arrive at the reconstruction time for the full dataset, this number is multiplied by the total number of slices and divided by four to correct for the number of GPUs. We see that TV-MIN is faster in each case, but that the relative difference diminishes substantially for larger datasets.

The TV-MIN and Noise2Inverse reconstructions of the static fuel cell are visually comparable. In the other two cases, the TV-MIN reconstruction suffers from residual noise and stair-casing artifacts, which diminish the visibility of fine details. These results confirm the findings in^25^, where it was found that Noise2Inverse could substantially outperform TV-MIN in terms of common image metrics. Image metrics cannot be computed in this case, because ground-truth images are not available.

## Discussion and conclusion

In this paper, we have shown that Noise2Inverse can be extended in space, time, and spectrum-like domains. As a result, it can be effectively applied to tomographic imaging techniques in common use at synchrotron X-ray facilities. In the case of static and dynamic micro-tomography, we have shown that Noise2Inverse offers significant improvements in the quality of denoising over alternative reconstruction methods. In the case of X-ray diffraction tomography, we have shown that the acquisition time can be substantially reduced while maintaining image quality.

Using the Noise2Inverse conditions, we have discussed the considerations that enter into successful application of the method to several synchrotron imaging techniques. As noted in^25^, the Noise2Inverse method benefits from high-resolution angular sampling, even at the cost of more measurement noise, but fares worse when angular sampling is sparse. We have shown that the effects of angular undersampling can be mitigated by exploiting interlaced angular sampling. We emphasize that Noise2Inverse does not assume a Gaussian noise model. The only assumption is that the noise does not result in a systematic upward or downward bias in the sinogram pixel intensities. Bias can result also from preprocessing, e.g., log correction and correcting for photon starvation, but for most realistic scenarios, the resulting bias is less than $1\%$.

Apart from measurement noise, vibrations and drifts of the sample constitute another substantial experimental uncertainty in dynamic micro-tomography. We note that the proposed approach does not deal with this issue directly: vibration of the sample can be visually identified between time steps in the Noise2Inverse reconstructions of the dynamic fuel cell dataset. It has been noted in previous work, however, that the improved visual reconstruction quality due to Noise2Inverse has enabled determining the correct center of rotation with greater precision^25^. Precise correction for sample vibration and drift as a preprocessing step is a topic for future work.

An open question is to what extent successful application to dynamic tomography depends on the similarity of the training time steps to later time steps. In our experiments, water was not present in the training data, but it was present in the rest of the sequence. We did not notice a deterioration of denoising accuracy for the water compared to other structures. This may be different in cases where the dynamics introduce more substantial changes in the sample structure. In general, we remark that the generalizability of the trained network mainly depends on the data that it is trained with and less so on whether it is trained in a supervised way or using Noise2Inverse.

The findings of this paper demonstrate numerous strong features of the Noise2Inverse method. First of all, its versatility was shown on both large and small datasets that were obtained using different imaging techniques. In this work, it was applied to a dynamic tomography dataset hundreds of gigabytes in size, and an XRD-CT dataset of less than $10 \hbox { MB}$. In addition, the method requires no CNN hyper-parameter tuning for training, which substantially simplifies its use in practice. We were able to achieve all results in this work using the same network architecture and the same CNN hyper-parameters, illustrating the sample-independence of the method. Finally, since the MS-D network architecture has a small number of parameters, it is unlikely to overfit to the noise. Therefore, prevention of overfitting through early stopping is not necessary, reducing the necessity for continuous observation of intermediate training results. All in all, the proposed method not only produces accurately denoised reconstructions, but can also be used in a “launch and forget” style, approaching the convenience of conventional reconstruction methods in a way that is uncommon for CNN-based methods. To scientists at synchrotron facilities, the sample-independence of the method could be appealing, as it opens up the possibility of blind application of the method to a variety of different samples.

## Supplementary information

Supplementary material 1 (pdf 1711 KB)

## Data Availability

The X-ray diffraction computed tomography dataset analyzed during the current study is publicly available via^52^. The raw fuel cell datasets are part of the TomoBank repository^46^.
