# Supplementary material for: Deep denoising for multi-dimensional synchrotron X-ray tomography without high-quality reference data
Source: Sci Rep. 2021 Jun 4;11:11895. doi: 10.1038/s41598-021-91084-8 (PMC8178391; doi:10.1038/s41598-021-91084-8)
Supplement: Supplementary file 1 — Supplementary material 1 (pdf 1711 KB) [file 41598_2021_91084_MOESM1_ESM.pdf]

Supplemental Materials: Deep denoising for multi-dimensional synchrotron X-ray tomography without high-quality reference data

Allard A. Hendriksen, Minna Bührer, Laura Leone, Marco Merlini, Nicola Vigano, Daniël M. Pelt, Federica Marone, Marco di Michiel, K. Joost Batenburg

S1 Data acquisition

X-ray tomography datasets were acquired at the TOMCAT beamline at the Swiss Light Source (SLS) at the Paul Scherrer Institut (PSI), Villigen, Switzerland. In addition, an X-ray diffraction tomography (XRD-CT) dataset was acquired at the ID15A beamline at the European Synchrotron (ESRF), Grenoble, France.

Static X-ray 3D micro-tomography The data was acquired at the TOMCAT beamline[1], and is publicly available[2]. The dataset contained 1001 projection images across an angular range of 180° measuring 1100×1440 square pixels of size $2.75\,\mu$m. The acquisition took 1 s, the exposure time was 1 ms, and the mean energy of the polychromatic beam was 30 keV.

Dynamic X-ray micro-tomography This dataset was acquired at the TOMCAT beamline[1], and is publicly available[2]. A full scan was performed every 0.1 s, resulting in a dataset of 180 time steps spanning approximately 32 seconds, divided into three 6 s chunks with 7 s pauses in between. Each time step contained 300 projection images across an angular range of 180° measuring 1100×1440 square pixels of size $2.75\,\mu$m. The mean energy of the polychromatic beam was 30 keV.

The documented angular increment was $\pi/300$ radians, consistent with the acquisition of 300 projection images per half rotation[2]. We observed a small deviation in this case. The angular increment was $\pi/299.924$, which could be determined up to five significant digits by visual inspection of the combined GridRec reconstruction of several time steps.

XRD-CT High-energy X-ray diffraction measurements were taken at the ID15A beamline using a monochromatic pencil beam (90 keV energy). Data was collected of 3 horizontal slices, spaced 7 mm apart, and acquisition of each slice took 20 minutes. Acquisition was performed in 273 translation steps over a scan range of 12 mm and in 225 rotational steps over an angular range of 180°. Sinograms were computed from the acquired images using the pyFAI library[3]. A subset of the sinograms was selected, containing 3 horizontal slices with 11 channels each. The displayed FBP reconstructions were computed with the Shepp-Logan filter using the ASTRA-toolbox[4].

S2 Noise2Inverse training

Network and hyper-parameters On each dataset, CNN training was performed using the same network architecture and the same hyper-parameters. An open source implementation of the MS-D network was used[5,6]. The networks were trained using the ADAM algorithm[7] with a mini-batch size of 24 and a learning rate of 10^{-3}. The networks had 100 single-channel intermediate layers, and the convolution in layer i was dilated by $d_i = 1 + (i \mod 10)$, as described in[5].

Static X-ray micro-tomography Noise2Inverse training was performed using the MS-D network architecture[5] with $54,796$ parameters. 2.5D-CNN training was performed using 10 context slices, of which 5 were below and 5 above the target slice. Reconstructions were computed using the GridRec algorithm[8,9], resulting in a rectangular reconstruction volume of $1100 \times 1440 \times 433$ voxels, containing the region of interest in which the fuel cell was located.

Dynamic X-ray micro-tomography Dynamic Noise2Inverse training was performed on the first 36 time steps of the experiments (3.6 seconds), in which the sample was stationary. The time steps were divided into 26 batches, each containing 6 time steps spaced one time step apart. The first batch contained time steps $1, 3, 5, 7, 9, 11$, the second batch contained time steps $2, 4, 6, 8, 10, 12$, et cetera. In this arrangement, the angles of the projection images were evenly divided over a 360° arc. Within each batch, the individual time steps served alternately as input, and the reconstruction of the combined remaining time steps served as the target. As in the static case, 2.5D-CNN training was used to supply an MS-D network with 10 context slices, and reconstruction was performed using the GridRec reconstruction algorithm.

XRD-CT Noise2Inverse training was performed using the MS-D network with 11 input channels and 11 output channels, resulting in $56,046$ parameters. Because the number of projection angles (225) was divisible by three, the sinogram was split in three parts in the angular domain. In each training iteration, two parts were used in the input reconstruction, and one part was used in the target reconstruction. We observed better performance using this scheme than by splitting the sinogram in two. During training, reconstructions were computed using FBP with the Ram-Lak filter[10] (instead of the Shepp-Logan filter) using the ASTRA-toolbox.

S3 Synthetic noise procedure for XRD-CT

We describe the procedure for applying synthetic noise to the original XRD-CT acquisition. In addition, we describe how we estimated the virtual acquisition time using noise estimates from the reconstructed images.

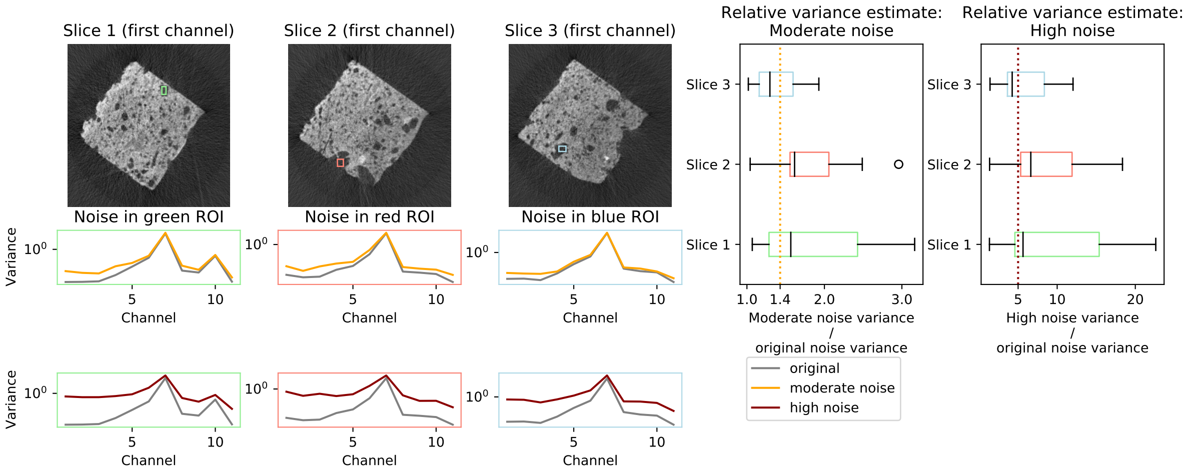

Figure S1. Estimating the noise level from reconstructed images (original, moderate synthetic noise, and high synthetic noise). In each of the three reconstructed slices, a region of interest (ROI) was chosen — indicated in green, red, and blue — that was uniformly dark in each of the 11 channels. In the left bottom panels, for each of these ROIs, the variance of the noise is plotted for the original, moderate noise, and high noise acquisition. In the right two panels, a box plot displays the uncertainty in the relative variance of the synthetic noise as it is divided by the variance in the original reconstruction. In the box plot, the vertical dotted lines show the conservative estimate of the relative variance, which was used to calculate the virtual acquisition time.

Synthetic noise was applied to the acquired sinograms. The noisy value of a pixel with intensity p at scattering angle θ was determined by adding Gaussian noise

$$p_{\text{noisy}} = p + \sqrt{\frac{|p|}{\theta \cdot T}}\mathcal{N}(0,1),$$

where $\mathcal{N}(0,1)$ is a unit-normal Gaussian random variable, and T is a fixed constant that determines the intensity of the noise. We used values $T = 1.0, 0.1$, corresponding to moderate and high noise, respectively. The Gaussian distribution is a good approximation of the noise in practice, due to the azimuthal integration, which averages multiple Poisson-distributed pixels.

We estimated the virtual acquisition time that corresponded to the applied noise. The variance of the noise follows an inverse linear relation with respect to the acquisition time: higher noise is associated with shorter acquisition times. Therefore, we picked three uniformly constant regions of interest (ROIs) in the three reconstructed slices for which we estimated the variance of the noise. This estimation was performed on the original reconstructions and the reconstructions with synthetic noise. The relative variance was estimated by dividing the variances in the ROIs with synthetic noise by the variance of the ROI in the original reconstruction. A box plot of the resulting estimates is shown in Figure S1. A conservative estimate yields that the variance of the moderate noise was 1.4 times higher than the original noise, and the variance of the high noise was 5 times higher than the original noise. This corresponds to an estimated virtual acquisition time of 70% and 20% of the original acquisition time, respectively.

S4 Total-variation minimization

The Total-Variation Minimization (TV-MIN) reconstructions were computed using the Chambolle-Pock algorithm[11], of which we used an open source implementation[1]. Because this implementation computes the full algorithm on the GPU, it is faster than comparable implementations.

On a single slice of the XRD-CT dataset (225 angles, 273 detector pixels), a reconstruction with 500 iterations takes roughly 1.7 seconds on a single GPU. The same reconstruction takes 184 seconds when computed on a single thread on the CPU with the widely used Tomopy package[9]. Assuming that work can be distributed over 32 threads, the CPU implementation would take 5.8 seconds per slice. This comparison was performed on a dual-socket system with Intel Xeon Silver 4110 CPUs clocked at 2.10GHz and four Nvidia GeForce GTX 1080 Ti GPUs.

Reconstructions were computed using various values of the regularization parameter λ on a regular exponential grid. These reconstructions are displayed in Figure S2. The chosen reconstruction is indicated in bold font.

[1] https://github.com/ahendriksen/ts_algorithms

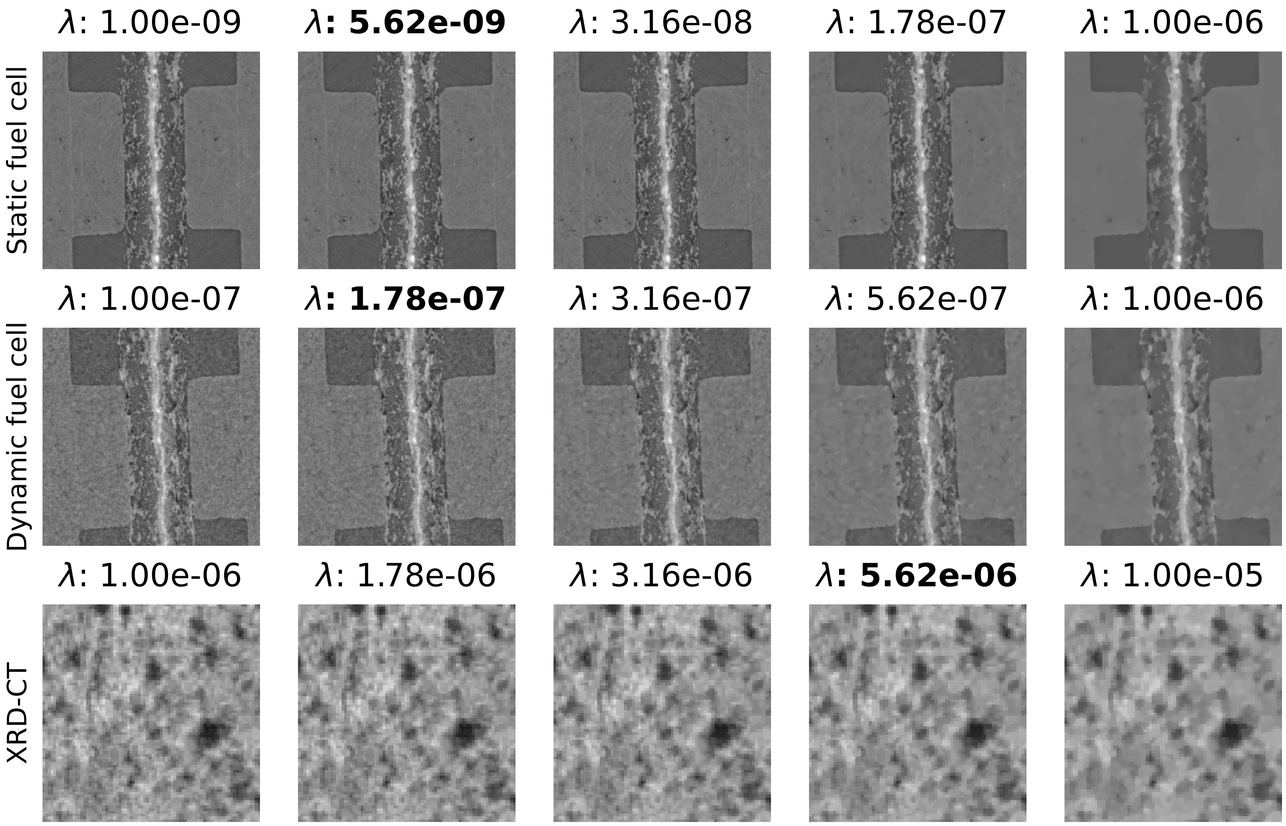

Figure S2. Total-variation minimization reconstructions with various values of the λ regularization parameter. Bold font indicates the chosen reconstruction that is used in Figure 7.

References

1. Bührer, M. *et al.* High-numerical-aperture macroscope optics for time-resolved experiments. *J. Synchrotron Radiat.* **26**, 1161–1172, DOI: 10.1107/s1600577519004119 (2019).
2. De Carlo, F. *et al.* TomoBank: a tomographic data repository for computational X-ray science. *Meas. Sci. Technol.* **29**, 034004, DOI: 10.1088/1361-6501/aa9c19 (2018).
3. Ashiotis, G. *et al.* The fast azimuthal integration python library: pyFai. *J. Appl. Crystallogr.* **48**, 510–519, DOI: 10.1107/s1600576715004306 (2015).
4. van Aarle, W. *et al.* Fast and flexible X-ray tomography using the ASTRA toolbox. *Opt. Express* **24**, 25129, DOI: 10.1364/oe.24.025129 (2016).
5. Pelt, D. M. & Sethian, J. A. A mixed-scale dense convolutional neural network for image analysis. *Proc. Natl. Acad. Sci.* **115**, 254–259, DOI: 10.1073/pnas.1715832114 (2017).
6. Hendriksen, A. ahendriksenh/msd_pytorch: v0.7.2, DOI: 10.5281/zenodo.3560114 (2019).
7. Reddi, S. J., Kale, S. & Kumar, S. On the convergence of ADAM and beyond. In *International Conference on Learning Representations* (2018).
8. Marone, F. & Stampanoni, M. Regridding reconstruction algorithm for real-time tomographic imaging. *J. Synchrotron Radiat.* **19**, 1029–1037, DOI: 10.1107/s0909049512032864 (2012).
9. Gürsoy, D., De Carlo, F., Xiao, X. & Jacobsen, C. Tomopy: a framework for the analysis of synchrotron tomographic data. *J. Synchrotron Radiat.* **21**, 1188–1193, DOI: 10.1107/s1600577514013939 (2014).
10. Buzug, T. M. *Computed Tomography : from Photon Statistics to Modern Cone-Beam CT* (Springer, 2008).
11. Sidky, E. Y., Jørgensen, J. H. & Pan, X. Convex optimization problem prototyping for image reconstruction in computed tomography with the Chambolle-Pock algorithm. *Phys. Medicine Biol.* **57**, 3065–3091, DOI: 10.1088/0031-9155/57/10/3065 (2012).
